# Effects of Virtual Reality versus Exercise on Pain, Functional, Somatosensory and Psychosocial Outcomes in Patients with Non-specific Chronic Neck Pain: A Randomized Clinical Trial

**DOI:** 10.3390/ijerph17165950

**Published:** 2020-08-16

**Authors:** David Morales Tejera, Hector Beltran-Alacreu, Roberto Cano-de-la-Cuerda, Jose Vicente Leon Hernández, Aitor Martín-Pintado-Zugasti, César Calvo-Lobo, Alfonso Gil-Martínez, Josué Fernández-Carnero

**Affiliations:** 1 Escuela Internacional de Doctorado, Department of Physical Therapy, Occupational Therapy, Rehabilitation and Physical Medicine, Universidad Rey Juan Carlos, 28032 Alcorcón, Spain; 2 Departamento de Fisioterapia, Facultad de Medicina, Universidad San Pablo-CEU, CEU Universities, 28008 Madrid, Spain; martinpintado@ceu.es; 3 Departamento de Fisioterapia, Centro Superior de Estudios Universitarios La Salle, Universidad Autónoma de Madrid, 28043 Madrid, Spain; hector.beltran@lasallecampus.es (H.B.-A.);; 4 CranioSpain Research Group, Centro Superior de Estudios Universitarios La Salle, Universidad Autónoma de Madrid, 28043 Madrid, Spainjosue.fernandez@urjc.es (J.F.-C.); 5 Department of Physical Therapy, Occupational Therapy, Rehabilitation and Physical Medicine, Rey Juan Carlos University, 28922 Madrid, Spain; roberto.cano@urjc.es; 6 Motion in Brains Research Group, Institute of Neuroscience and Sciences of the Movement (INCIMOV), Centro Superior de Estudios Universitarios La Salle, Universidad Autónoma de Madrid, 28043 Madrid, Spain; 7 Facultad de Enfermería, Fisioterapia y Podología, Universidad Complutense de Madrid, 28040 Madrid, Spain; cescalvo@ucm.es; 8 Unit of Physiotherapy, Hospital La Paz Institute for Health Research, IdiPAZ, 28029 Madrid, Spain; 9 Grupo Multidisciplinar de Investigación y Tratamiento del Dolor. Grupo de Excelencia Investigadora URJC-Banco de Santander, 28922 Madrid, Spain

**Keywords:** exercise, neck pain, chronic pain, virtual reality, virtual reality exposure therapy

## Abstract

*Background*: Virtual reality (VR) applied to patients with neck pain is a promising intervention to produce positive effects when used alone or combined with exercise. Therefore, the objective of this manuscript is to compare the effects of VR versus exercise treatment on pain intensity, conditioned pain modulation (CPM), temporal summation (TS) and functional and somatosensory outcomes in patients with non-specific chronic neck pain (NS-CNP). *Methods*: A single-blinded, randomized clinical trial was carried out. A total sample of 44 patients with NS-CNP was randomized into a VR treatment group or neck exercises group. The intervention consisted of two treatment sessions per week, for four weeks and eight sessions. Four measurement moments (at baseline, immediately, 1 month, and 3 months after intervention) were considered. Pain intensity, CPM, TS, functional and somatosensory outcomes were measured. *Results*: Statistically significant differences were revealed for time factor (F = 16.40, *p* < 0.01, η_p_^2^ = 0.28) and group*time interaction for kinesiophobia (F = 3.89, *p* = 0.01, η_p_^2^ = 0.08) showing post-hoc differences in favor of the VR group at 3 months (*p* < 0.05, d = 0.65). Significant effects were shown for time factor (*p* < 0.05) but not for the group*time interaction (*p* > 0.05) for pain intensity, rotation range of motion (ROM), Neck Disability Index, pain catastrophizing, fear-avoidance beliefs, left side pressure pain threshold (PPT) and anxiety. Statistically significant differences were not found for time factor (*p* > 0.05) and neither in group*time interaction (*p* > 0.05) for CPM, TS, right side PPT, flexo-extension and lateral-flexion ROM. *Conclusions*: Kinesiophobia was the only outcome that showed differences between VR and exercise at 3 months. Nevertheless, pain intensity, CPM, TS, ROM, neck disability, pain catastrophizing, fear-avoidance beliefs, PPT and anxiety did not show differences between both interventions.

## 1. Introduction

Between 50% and 85% of the general population experience neck pain at some point in their lives and every person who experiences neck pain is likely to experience it again 1–5 years later [1]. A recent study [2] showed that the prevalence, incidence and years lived with disability from 1990 to 2017 has not changed significantly from the previous report [3]. The global prevalence was 3.5%, 288.7 million cases, from which 65.3 million cases were incident and 28.6 million spent years living with a disability. The prevalence of neck pain in Spain increased from 7.86% to 8.56% between 2009 and 2012 [4].

There is a strong relationship between cervical disability and neck pain [5,6]. Within the functional alterations, the active range of motion (ROM) is an important physical variable for daily life activities and it aids in stabilizing and orientating the head [7]. It has been proven that the cervical ROM decreases in patients who have neck pain [7,8,9], which may be related to the degree of pain and cervical functionality [8,9,10,11].

In addition, psychological factors, such as fear of movement, anxiety or depression, are also associated with chronic neck pain [12,13]. These psychological factors trigger a functionality decrease, which is explained by the “fear-avoidance model”, associated with increased hypervigilance, leading to a vicious cycle of psychological and physical factors that lower the threshold at which painful experiences are triggered [14,15,16]. High levels of fear of movement may be correlated with a decrease in the cervical ROM and/or speed of cervical movement. Neck disability is also positively correlated with pain catastrophizing in patients with both chronic neck pain and cervico-craniofacial pain, contributing to the intensity of pain and the progression of the disorders [17].

Moreover, alterations of pain processing have been investigated in patients with neck pain, showing local and distant mechanical hyperalgesia [18]. Regarding nociceptive processing alterations, other researchers have found that some subgroups of patients with chronic neck pain present generalized and bilateral hyperalgesia in addition to local hyperalgesia in the cervical region, as well as functional alterations such as decreased range of cervical movement [19,20,21]. Even in some subgroups, we have found that generalized pain is associated with psychosocial factors such as anxiety [20]. 

Regarding the management of neck pain and its related alterations, attentional distraction—defined as shifting the attention away from the pain—is a commonly used strategy. In this context, virtual reality (VR) is defined as a computing system used to create an artificial world in which users have the impression of being able to navigate and manipulate objects in it [19,20]. VR treatment is based on three basic elements: simulation, interaction and immersion. Thanks to these treatments, the patient is able to interact intuitively by perceiving visual, auditory, tactile or kinesthetic stimuli in a virtual world [21]. Therefore, VR treatment offers increased feedback, turning the technique into a powerful pain-distracting mechanism. It has been used in burn patients, central sensitization, chronic pain and neurological disorders [22,23]. Interaction and immersion in VR systems determine the different types of VR (immersive, semi-investment projection, semi-investment second person, non-immersive or video games). Among these, immersive VR could be used in patients with neck pain as a low-cost therapeutic measure in the treatment of chronic neck pain, due to its adaptability and its distracting component [24,25].

The hypoalgesic effects of distraction by VR have been mainly investigated during experimentally induced pain in healthy subjects or during acute procedural pain. In rehabilitation sciences, some clinical trials have previously evaluated the effectiveness of VR on individuals with neck pain [22,23]. Two high-quality studies found statistically significant improvements in the range of neck flexion, speed, the precision of movement, quality of life, pain intensity and neck disability with VR compared to control with kinematic training with an over-the-head laser and compared to an untreated group [22,23]. Nevertheless, findings from these studies were obtained by using the same VR system to measure neck function, so the previous training of the patients could have influenced the results [24].

Although other studies have failed to demonstrate a hypoalgesic effect of distraction, the majority of studies have shown that distraction does reduce pain, most of them in acute conditions [25]. A meta-analysis published in 2019 [26] showed that VR applied to patients with neck pain was promising in producing positive effects when applied alone or combined with exercises. 

Therefore, the main objective of this study is to compare the effects of VR versus exercise on pain intensity as well as conditioned pain modulation (CPM) and temporal summation (TS) in patients with non-specific chronic neck pain (NS-CNP). Secondly, we aim to evaluate the effects of VR versus exercise on functional and psychological variables in patients with NS-CNP.

## 2. Materials and Methods

### 2.1. Study Design

A randomized single-blind controlled trial was conducted in conformity with the Consolidated Standards of Reporting Trials (CONSORT) [27] requirements and approved by the ethics committee of the Rey Juan Carlos University. The study was registered in the United States Randomized Trials Register on clinicaltrial.gov (NCT04265248).

### 2.2. Participants

From January 2020 to March 2020, 44 individuals with NS-CNP were recruited from Rey Juan Carlos University, CEU San Pablo University, and the Community of Madrid through social networks, posters, brochures and emails. The inclusion criteria were: (a) non-specific chronic neck pain; (b) age 18 to 65 years. The exclusion criteria were the following: (a) pregnancy; (b) specific neck pain caused by metastasis, neoplasia, infectious or inflammatory disorders, bone fractures or traumatic precedents with neck injuries; (c) positive neurological signs or evidence of spinal compression (abnormal diffuse sensitivity, hyperreflexia, or diffuse weakness); (d) cervical osteoarthritis; (e) spondyloarthritis; (f) neck pain associated with vertigo (vestibular involvement); (g) neck pain associated with whiplash injuries; (h) previous cervical surgeries; (i) headaches prior to the onset of neck pain and without cervical origin; and (e) inability to provide informed consent. 

All participants received an explanation of the study procedures, which were planned according to the ethical standards of the Declaration of Helsinki [28]. Written informed consent was obtained from all participants before their inclusion.

### 2.3. Research Team Settlement

Two trained physical therapists were assigned to provide the treatments and collect the data from each subject, including pain related measurements and psychological variables. A different researcher conducted the statistical analysis and other assessors assisted in writing and reviewing the document. 

Only the person in charge of the statistical analysis was blinded. Neither the therapists or the patients were blinded due to the nature of the proposed experiment.

### 2.4. Interventions

#### 2.4.1. Virtual Reality Treatment

**Figure 1:**
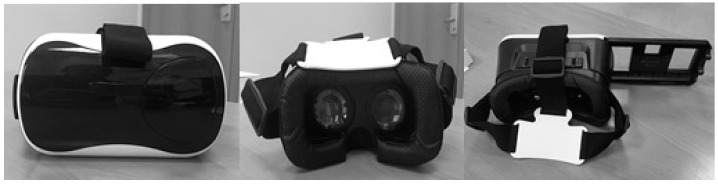
VR Vox Play virtual reality glasses.

**Figure 2:**
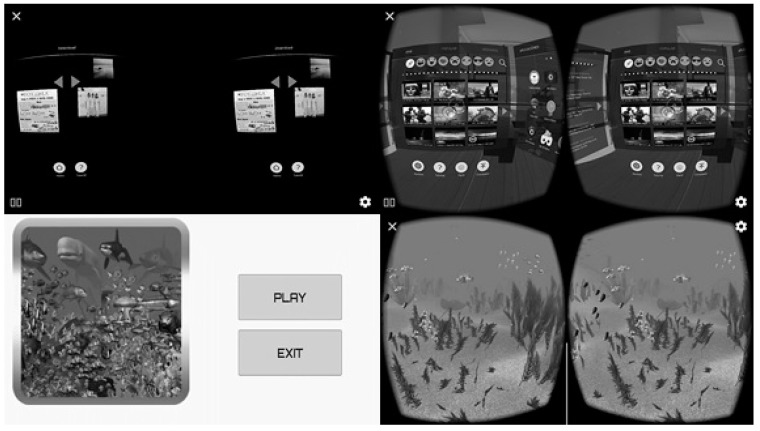
Virtual reality mobile applications: “Full Dive VR” on the top and “VR Ocean Aquarium 3D” on the bottom.

The VR Vox Play glasses were used with an HMD clamping system (weight 330 g) to which a Smartphone (LG Q6) was attached (Figure 1). Two VR mobile applications were installed. The first was a "Fulldive VR" as the first degree of difficulty for the participants, where only tilting movements of the neck are necessary. As a second degree of difficulty, the game “VR Ocean Aquarium 3D” was used, where flexion, extension and rotation movements must be integrated in addition to introducing a sensory element by integrating the sound of the sea (Figure 2).

The subjects started with the free mobile application “Fulldive VR” in which only lateral flexion movements are necessary. The patient was immersed in an environment that simulated the living room of a house and at the same time they could visualize a gallery of previously selected photos. Then, they had to change the images in the viewfinder by tilting their neck bilaterally, as well as naming the photos that were displayed. New images were added each week to introduce a novel factor to the patient and to keep them motivated by the exercise program. Once the patient felt comfortable with the system and adapted to the headset, then after one minute rest, the difficulty of the exercise program was increased with the free mobile application “VR Ocean Aquarium 3D” where flexion, extension and rotation movements were added. The patient was immersed in a virtual environment that simulated an ocean, moving forward and observing different marine animals by making the neck movements. The patient named the animals they were visualizing. In addition, the application presented auditory and sensory stimuli by integrating the sound of the sea.

In the first sessions, the patients were instructed to perform a previous cranio-cervical flexion before starting any movement. Then, the therapist controlled the contraction of the superficial musculature with their hands. Gradually, the aid of the physiotherapist was removed so that the participants could integrate deep muscle contraction and the correct cranio-cervical posture innately. For these patients to carry out the same work as the control group, the physiotherapist counted and controlled in each exercise the number of movements the patient performed, in order to not exceed the dose proposed: 3 series of 10 repetitions of each exercise with 30 s rest between exercises.

#### 2.4.2. Neck Exercises

**Figure 3:**
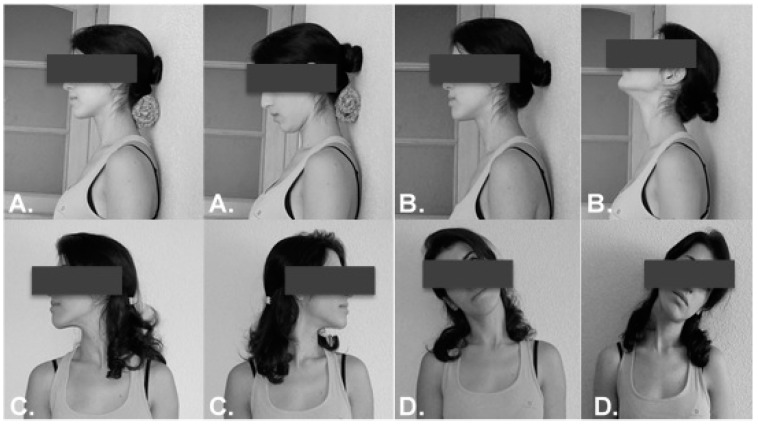
Neck exercises. (**A**) Flexion; (**B**) extension; (**C**) rotation; (**D**) lateral flexion.

This group performed three series of 10 repetitions of every exercise, with a 30 s rest between exercises. The researcher provided the necessary verbal corrections for the proper execution of the exercises, using the same verbal commands for all participants (Figure 3). Flexion exercise: in a sitting position, a ball was placed between the wall and the neck of the patient that performed a controlled neck flexion with a previously maintained cranio-cervical flexion. Extension exercise: in a sitting position, the participant was asked to perform a controlled neck extension and a cranio-cervical flexion before returning to the initial position. Rotation and tilt exercises: in a sitting position, the subject who had previously done a cranio-cervical flexion to activate the deep flexor musculature was asked to perform the movement.

### 2.5. Outcome Measures

#### 2.5.1. Primary Outcomes

##### Visual Analog Scale (VAS)

This scale created by Scott Huskinsson in 1976 [29] was used to measure pain intensity and its evolution. It consists of a 100 mm horizontal or vertical line, the left end represents “no pain” and the right represents the “worst pain imaginable” in which the patient must make a mark to indicate the intensity of their pain [30]. The assessment is established as mild pain if the patient scores pain less than 30 mm, moderate if the assessment is between 31 and 54 mm, and 55 mm and above is classified as severe pain [31]. A difference in pain severity of 30 mm or greater is considered to be a clinically important change [32]. Its validity and reliability have been demonstrated in previous studies [33,34,35].

##### Conditioned Pain Modulation 

CPM is a psychophysical experimental measure of the endogenous pain inhibitory pathway. The CPM test consists of the evaluation of a painful test stimulus followed by a second evaluation at the same time as a distant, painful conditioning stimulus (parallel paradigm). The test stimulus was a pressure pain threshold (PPT) measured using a 1 cm^2^ tipped-diameter algometer (Force Ten TM FDX Digital Force Gauge) applied at a rate of 40 kPa/s [36]. 

CPM was then induced by combining the protocol described by Cathcart et al. and an inflated occlusion cuff around the subject’s arm, contralateral to the most affected trapezius, until the patient felt a painful intensity (conditioning stimulus) of 6/10. The occlusion cuff was inflated at a rate of 20 mm Hg/s and held for 30 s. After another 30 s, the pressure was made on the distal phalanx of the first finger on the opposite hand until the patient noticed a painful sensation. The cuff pressure was then increased or decreased until the intensity of the pain was rated 3/10.

This method has been shown to have good reliability and validity for examining the temporal sum and CPM [37].

##### Temporal Summation

TS consisted of the application of a series of short noxious stimuli which typically results in a continuously increasing perception of pain along with the series. This represents features of central sensitization of pain pathways, equivalent to the wind-up physiological phenomenon [38]. The protocol described by Cathcart et al. was used to measure the TS, which are established ways to measure the excitability of nociceptor pathways and descending pain inhibition, respectively.

Initially, a PPT used as a test stimulus was measured locally and distally. Then, the TS was triggered by 10 consecutive pressures on the PPT at the pressure determined at each location. For each pulse, the pressure was gradually increased at a rate of 2 kg/s at the determined PPT and held for 1 s before being released (with an interval of one second between each stimulus). The intensity of the 1st, 5th and 10th pulse pain was assessed using a numerical rating scale (0 being no pain and 10 being worst possible pain). A 5 min rest period was programmed afterward [37].

#### 2.5.2. Secondary Outcomes

##### Active Cervical Range of Movement

To assess the active cervical range of movement (CROM) the CROM device (Performance Attainment Associates, Lindstrom, MN, USA) was used, which is composed of two goniometers that allow measuring the range of motion in the different planes of movement [39]. Each movement is recorded three times with a pause in between, and the average of the three is used for the statistical analysis [40]. 

This device has been verified to be reliable for measuring cervical ROM (r = 0.98; 95% CI 0.95–0.99) compared to the universal, gravitational goniometer or visual estimation. In addition, it has a lower cost than other more technological systems to take cervical measurements such as the 3D Space System or radiographic measurements [41].

##### Neck Disability Index (NDI)

Neck Disability Index is a modification of the Oswestry Disability Index and is the most widely used scale for cervical dysfunction and pain. In addition, it is a one-dimensional measure that is used to determine the level of disability that the patient perceives due to neck pain [42,43]. It is a self-questionnaire which consisted of 10 items on activities of daily living. Each section is scored from 0 (absence of disability) to 5 (complete disability). Most studies suggest that this scale has acceptable reliability, although the intra-class correlation coefficients (ICC) range from 0.50 to 0.98 [43,44,45].

##### Pain Catastrophizing

The Pain Catastrophizing Scale assesses the degree of negative thoughts and feelings about patients’ pain. It consists of 13 items valued from 0 (never) to 4 (always). The possible range is 0–52 points, with lower scores indicating less catastrophism levels [46]. It is a reliable tool with a Cronbach’s α value greater than 0.70 [47].

##### Pain-Related Fear of Movement/(Re)Injury

The Pain-related fear of movement was assessed using the 11-item Spanish Version of the Tampa Scale of Kinesiophobia [48]. The final score can range between 11 and 44 points, with higher scores indicating greater perceived kinesiophobia. This scale has been shown to have good validity and reliability with a Cronbach’s α of 0.79 in a sample of chronic pain [49].

##### Fear-Avoidance Beliefs

The fear-avoidance beliefs questionnaire (FABQ) was developed to measure levels of fear and avoidance beliefs about work and physical activity. The instrument consists of two subscales, a four-item physical activity subscale (FABQ-PA) and a seven-item work subscale (FABQ-W). Each item is scored from 0 to 6 and summed to produce the subscale score. Possible scores range from 0–28 to 0–42 for the FABQ-PA and FABQ-W, respectively [50]. This scale has shown to have good validity and reliability with a Cronbach’s α of 0.93 [51].

##### Pain Pressure Thresholds

The pain pressure threshold (PPT) is defined as the minimum pressure at which the pressure sensation changes to a pain sensation. The mechanical pressure algometer (Force Ten TM FDX Digital Force Gauge) employed in this study consisted of a round rubber disk (area of 1 cm^2^) attached to a pressure (force) gauge. The pressure was applied at a rate of 0.31 kg/s [52].

Prior to the assessment, the skin regions overlying the upper trapezius muscle and the tibialis anterior were marked with a pen. During the measurements, the algometer was held perpendicular to the skin and the evaluator made the pressure. The participants were then told to immediately alert the evaluator when the pressure became painful, at which point the mechanical stimulus was stopped. The data used for the statistical analysis consisted of three consecutive measurements of the PPT at the two locations using the mean of these measurements. The reliability of pressure algometry is high (intra-class correlation coefficient [ICC], 0.91; 95% confidence interval [CI] 0.82–0.97) [52].

##### Pain-Related Anxiety

Pain-related anxiety was measured with the 20 items version of the Pain Anxiety Symptoms Scale (PASS-20), described by McCracken and Dhingrain in 2002. It consists of 20 items that measure four components of pain anxiety: fear, escape/avoidance, cognitive anxiety and physiological anxiety. Patients score each of these items through a six-point Likert-type scale, in which 0 is never and 5 is always. Total score ranges from 0 to 100 with higher scores indicating greater anxiety about pain [53,54]. The PASS-20 has good psychometric properties with adequate internal consistency and concurrent validity. Cronbach’s α was obtained in a range of 0.91 to 0.92 [55,56]. The Spanish validated version of the PASS-20 also has good psychometric properties with good internal consistency (Cronbach’s α of 0.93) [57].

### 2.6. Procedure

Before entering the study and prior to the intervention, all participants granted their consent and were given a set of questionnaires which included self-reported psychometric forms to evaluate the psychological outcomes and sociodemographic data. Subsequently, the pre-intervention measurements were performed and depending on the group they belonged to according to the randomization, the participants performed the VR intervention or the neck exercises. The intervention consisted of two treatment sessions per week for 4 weeks, with eight sessions in total. This study showed a 3 months follow-up with evaluations at baseline, immediately, 1 month and 3 months after the intervention. The interventions and the CROM measurement were performed in the sitting position while the PPT, TS and CPM measurements were measured in the supine position. 

### 2.7. Sample Size Calculation and Randomization

The Visual Analogue Scale (VAS) was selected as the primary measurement of our results in this study. The effect of the size for the VAS was estimated in medium (SE = 0.25). The correlation between repeated measures was assumed to be 0.5. Assuming the performance of four measurements (prior to the intervention, after one week, one month and three months), the sphericity correction was determined to be 0.5. With a statistical power of 0.80 and an alpha level of 0.05, a total sample size of 36 patients was estimated. Taking into account 15% of the losses, it was necessary to reach a total of 42 patients. The sample size was calculated using the program G*Power 3.1.7 (G*Power from University of Düsseldorf, Düsseldorf, Germany) [58]. 

The randomization was performed using a computer-generated random sequence table with an unbalanced four-block design (GraphPad Software Inc., San Diego, CA, USA). One of the researchers that was not involved in the data collection or intervention of the subjects of the study generated and oversaw the randomization list. A number was randomly given to each of the subjects and with the software they were anonymously and randomly allocated in one of the two groups using the generated list, ensuring the blind allocation and including 22 participants in each group. Nonetheless, the subjects were conscious of whether or not they were wearing the VR headset therefore they knew if they belonged to the intervention or control group and could not be blinded. Qualified physiotherapists performed the assessments and interventions and a different researcher who was blinded to the allocation conducted the assessment of the outcomes.

### 2.8. Statistical Analysis

Data analysis was carried out using the version 25.0 of the SPSS statistical program (IBM, Armonk, NY, USA). The Shapiro–Wilk test was applied to check the fit of the variables to a normal distribution. The results of the study were represented using descriptive statistics (mean, standard deviation and route for the parametric variables, median or mode, ranges and quartiles for the non-parametric variables). For comparisons between groups, a simple analysis of variance (ANOVA) or mixed variance models (2 × 4) with post hoc Bonferroni tests for multiple comparisons were applied. 

For analysis of the variable temporal summation, we used non-parametric statistics because it presented an abnormal distribution. Descriptive statistics were used to summarize data, including means and SDs, medians and interquartile ranges (IQR) for continuous data. The Kruskal–Wallis test was used to compare the two groups to baseline data and across groups each time. The Friedman test was used to analyze the change from the intragroup results and the Wilcoxon signed-rank test was used for post hoc intragroup comparisons. 

For the data analysis, a 95% confidence interval was used. All values that had a *p* < 0.05 were considered statistically significant. Partial eta squared was calculated for the parametric variables, meanwhile rank correlation was calculated for the non-parametric on the primary and secondary outcome variables. The magnitude of the effect was classified as small (0.20 to 0.49), medium (0.50 to 0.79) or large (0.8) according to Cohen’s method [59]. Analyses were performed according to the intention-to-treat principle. 

## 3. Results

**Figure 4:**
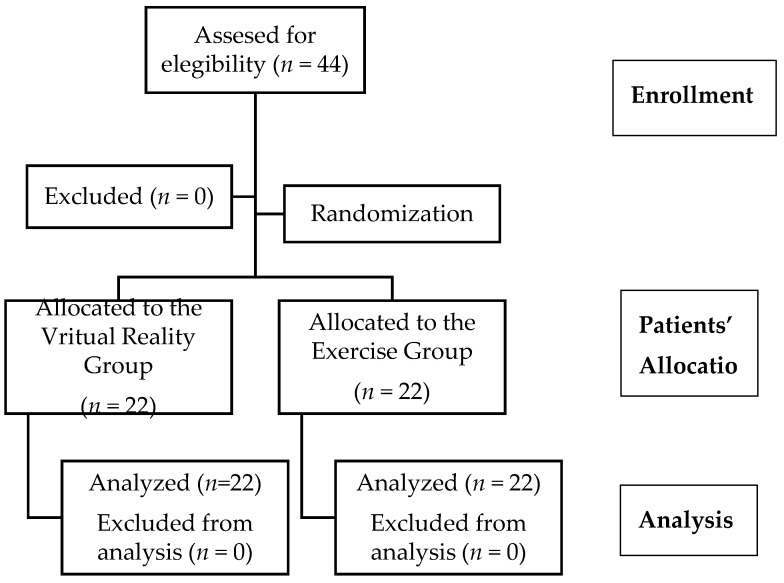
Recruitment flow diagram.

A total of 44 patients with NS-CNP with an average age of 29.7 ± 10.81 years, 21 males (47.7%) and 23 females (52.3%), were admitted after fulfilling the inclusion criteria from January to March 2020. Figure 4 shows the recruitment flow diagram of patients through the trial. The baseline characteristics of the patients in each group are presented in Table 1. No differences were found between groups at baseline.

### 3.1. Variables

#### 3.1.1. Neck Pain Intensity

Lineal general model showed significant effects for time factor (F = 32.06; *p* < 0.01; η_p_^2^ = 0.44) but not for the group*time interaction (F = 1.98; *p* = 0.12; η_p_^2^ = 0.47) for pain intensity over the VAS. The post hoc analysis revealed significant within-group differences in all groups between baseline and post-treatment and 1 month and 3 month follow-up measures. There was a large effect size for the VR group post-treatment (*p* = 0.01, *d* = 0.1.21), 1 month follow-up (*p* < 0.01, d = 1.12) and 3 month follow-up (*p* < 0.01, d = 1.44). There was a large effect size for control group post-treatment (*p* < 0.01, d = 0.82), 1 month follow-up (*p* < 0.01, d = 1.53) and 3 months follow-up (*p* < 0.01, d = 1.44) (Table 2).

#### 3.1.2. Conditioned Pain Modulation

No statistically significant differences were found for time factor (F = 1.135, *p* = 0.33, η_p_^2^ = 0.20) and neither in group*time interaction (F = 0.33, *p* = 0.751, η_p_^2^ = 0.08) (Table 2).

#### 3.1.3. Temporal Summation

In the Kruskal–Wallis test, differences were found for temporal summation at 1 month (*p* < 0.05)**.** The Wilcoxon test did not show significant differences when the baseline data were compared with the follow-up periods measured (*p* > 0.05). The Friedman test was not statistically significant (*p* > 0.05) for any group. All data can be observed in (Table 2).

#### 3.1.4. Range of Motion

For rotation movement, ANOVA revealed significant differences over time (F = 4.23, *p* < 0.01, η_p_^2^ = 0.09) for the rotation but not in the group*time interaction (F = 0.49, *p* = 0.63, η_p_^2^ = 0.01). There were no statistically significant effects for flex-extension and lateral flexion movements found in the time factor (F/E ROM F = 0.32, *p* = 0.71, η_p_^2^ < 0.01; lateral flexion F = 0.86, *p* = 0.46, η_p_^2^ = 0.02), nor for time*group interaction (F/E ROM F = 0.03, *p* = 0.99, η_p_^2^ < 0.01; lateral flexion F = 1.65, *p* = 0.18, η_p_^2^ = 0.04 ). The post hoc analysis was not significant for within-group differences in both groups between baseline and follow up measures for rotation motion (Table 2).

#### 3.1.5. Neck Disability Index

The ANOVA showed significant differences over time (F = 25.5, *p* < 0.01, η_p_^2^ = 0.38) but not in the group*time interaction (F = 0.02, *p* = 0.99, η_p_^2^ < 0.01) in the neck disability index. Table 2 shows the comparative results for each of the groups. The post hoc analysis revealed significant within-group differences in both groups between baseline and post-treatment, 1 month and 3 month follow-up measures, with a large effect size for VR group post-treatment (*p* = 0.01, d = 0.99), 1 month follow-up (*p* < 0.01, d = 1.20) and 3 month follow-up (*p* < 0.01, d = 1.35). There was a large effect size for control group post-treatment (p < 0.01, d = 0.87), 1 month follow-up (*p* < 0.01, d = 1.06) and 3 months follow-up (*p* < 0.01, d = 1.12) (Table 2).

#### 3.1.6. Pain Catastrophizing

In this psychological variable, the ANOVA showed significant differences over time (F = 25.20, *p* < 0.01, η_p_^2^ = 0.38) but not in the group*time interaction (F = 1.21, *p* = 0.29, η_p_^2^ = 0.03). The post hoc analysis revealed significant within-group differences in both groups between baseline and post-treatment, 1 month and 3 month follow-up measures, with a medium–large effect size for the VR group post-treatment (*p* < 0.01, d = 0.77), 1 month follow-up (*p* < 0.01, d = 0.97) and 3 month follow-up (*p* < 0.01, d = 1.19). There was a medium–large effect size for the control group post-treatment (*p* = 0.02, d = 0.70), 1 month follow-up (*p* < 0.01, d = 1.10) and 3 month follow-up (*p* = 0.02, d = 0.79). Table 2 shows the comparative results for each of the groups.

#### 3.1.7. Pain Kinesiophobia

The ANOVA revealed significant differences over time (F = 16.40, *p* < 0.01, η_p_^2^ = 0.28) and in the group*time interaction (F = 3.89, *p* = 0.01, η_p_^2^ = 0.08). The post hoc analysis revealed significant within-group differences in the VR group between baseline and follow-up at 3 months, with a medium effect size (*p* = 0.020, d = 0.67) The post hoc analysis revealed significant within-group differences only in the VR group between pre- and 1 month (*p* = 0.001, d= −0.90) and pre- and 3 months (*p* = 0.001, d = −1.42). Table 2 shows the comparative results for each group.

#### 3.1.8. Fear-Avoidance Beliefs

The ANOVA revealed significant differences over time (F = 19.70, *p* < 0.01, η_p_^2^ = 0.33) but not in the group*time interaction (F = 2.02, *p* = 0.115, η_p_^2^ = 0.04). Table 2 shows the comparative results for each of the groups. The post hoc analysis revealed significant within-group differences in all groups between baseline and 3 months follow-up- measures, with a large effect size for the VR group (*p* < 0.01, d = 1.06) and with medium effect size for the control group (*p* < 0.01, d = 0.66) (Table 2).

#### 3.1.9. Pressure Pain Threshold

The ANOVA showed statistically significant effects for time factor in the left side PPT (F = 7.07, *p* < 0.01, η_p_^2^ = 0.15) but no statistically significant effects were found for right side PPT (F = 2.20, *p* = 0.12, η_p_^2^ = 0.05). No statistically significant differences were found for either measure in the group*time interaction (PPT right F = 0.16, *p* = 0.92, η_p_^2^ = 0.00; PPT left F = 1.06, *p* = 0.35, η_p_^2^ = 0.02). The post hoc analysis revealed significant within-group differences in the VR group between baseline and 1 month and 3 month measures, with a medium effect size for PPT in the left side (1 month: *p* = 0.002, d = −0.67; 3 months: *p* = 0.02, d = −0.54; Table 2).

#### 3.1.10. Anxiety

In this variable, the ANOVA showed significant differences over time (F = 24.18, *p* < 0.01, η_p_^2^ = 0.37) but not in the group*time interaction (F = 0.64, *p* = 0.58, η_p_^2^ = 0.01). The post hoc analysis revealed significant within-group differences in all groups between baseline and post-treatment, 1 month and 3 month follow-up-intervention measures, with a medium–large effect size for the VR group post-treatment (*p* < 0.01, d = 0.54), 1 month follow-up (*p* < 0.01, d = 0.70) and 3 month follow-up (*p* < 0.01, d = 0.82). There was a medium effect size for the control group post-treatment (*p* < 0.01, d = 0.60), 1 month follow-up (*p* < 0.01, d = 0.72) and 3 months follow-up (*p* < 0.01, d = 0.75). Table 2 shows the comparative results for each of the groups.

## 4. Discussion

The main objective of this article was to compare the effects of immersive VR on pain intensity, CPM and TS versus evidence-based exercises in participants with NS-CNP. The second objective was to evaluate the effects of this experimental intervention on functional, somatosensory and psychosocial variables after VR treatment compared to the exercise group. Our results showed significant differences for both groups over time but not in the group*time interaction for NDI, temporal summation, PPT, fear-avoidance beliefs, catastrophizing, anxiety or cervical range of motion. The post hoc data analysis showed intra-group statistically significant differences in the VR group for PPT, NDI, kinesiophobia and pain catastrophizing, but not for ROM, temporal summation or conditioned pain modulation. Only kinesiophobia showed significant differences in the group*time interaction. These differences between groups were relevant after 3 months when patients in the VR group showed lower levels of kinesiophobia compared to those in the exercise group.

Our primary variables (VAS, CPM and TS) did not show statistically significant changes between groups at 3 months follow-up, meaning that VR can be as effective in modulating pain and decreasing perception to repetitive painful stimulation as evidence-based exercises for the neck. We found statistically significant changes in the TS for the intervention group at 1 month; however, there is little evidence and research that focuses on these variables related to chronic neck pain to compare our findings related to VR and chronic neck pain. Hayashi et al. in 2019 [60], found statistically significant changes in CPM and PPT of the quadriceps and forearm on healthy subjects after using VR with a head-mounted display. These authors hypothesized that benefits occurred via pain distraction mechanisms and reduced pain-related brain activity in the anterior cingulate cortex, primary and secondary somatosensory cortex, insula and thalamus. Moreover, it has been postulated that VR distracting effects originate from intercortical modulation among signaling pathways of the pain matrix trough attention, emotion, memory and other senses activated in VR environments [61]. 

Related to pain intensity, a recent meta-analysis concluded that immersive VR has minimal carryover effect beyond the immediate post VR exposure [62], which contradicts our findings. Nevertheless, other studies have found substantial changes that support our outcomes [25,63,64]; Lin et al. in 2019 found that VR has a significant effect on pain relief, motor function and joint mobility for patients with chronic musculoskeletal disorders [65]. Another new meta-analysis found that for patients with chronic neck pain there were statistically significant changes in function, pain intensity and disability at intermediate-term follow up [24]. In our research, only evidence-based exercises were included. Future studies should compare VR with other physical therapy treatment modalities.

Our secondary variables showed that VR could increase the range of motion for the rotation and decrease the perception of disability. We found in a previous study that the observed actions are also effective in improving the CROM. This may be due to the fact that the visualization of images might cause increased activity of the corticospinal system [66] and produce more intense muscle recruitment and ROM as a result. Despite the fact that changes in the CROM require further research, Hardy et al. in 2020 theorize that during a usually painful magnitude of movement, understated visual feedback may provide a strong safety cue that is sufficient to turn a painful movement into a pain-free one [67]. In 2019, Martinez et al. also found changes suggesting that observation of actions is stronger than motor imagery in increasing the CROM and the cervical joint position sense [68]. Our results on neck disability are also consistent with other studies. VR seems to show good short term effects on self-perceived disability especially on the post-treatment measurements, which is not unusual in intervention studies in neck pain [23].

These results may be explained when a patient’s chronic neck pain is deeply engaged in an immersive VR experience, as it becomes difficult to perceive stimuli outside of the field of attention. This makes pain management possible by blocking sensory information via opioid analgesics or by creating a distraction [69,70] after treatment periods are maintained over time.

The psychosocial variables also showed significant changes for both groups, especially in the VR group. For the fear-avoidance beliefs, in 2019 Fowler et al. [13] indicated that the use of VR on elderly people with a fear of movement showed positive results when the treatment matched the patient’s level of functioning. There was a progression in intensity so that the patients presented a positive perception which presented better benefits. Based on the results of this study, VR was only effective in reducing fear of movement in patients with NS-CNP. The improvement observed at 4 months in the VR group was higher than 10 points compared to the baseline, so it was superior to the minimal detectable change of 5.6 points previously reported. This suggests that VR interventions could be selected over exercise in patients with NS-CNP associated with higher levels of kinesiophobia [71].

Regarding kinesiophobia, we found that it was the only variable to have significantly improved compared to the exercise group, especially at 3 months follow-up, and was considered as an effective tool for reducing fear of movement. Other studies have found that VR reduces kinesiophobia in patients with chronic spinal pain which was associated with an increase in function [13,67,72]. In 2020, Martínez et al. concluded on a narrative review that the participants’ physical condition and cognitive and emotional characteristics should be considered before implementing interventions that employ movement representation techniques. They also found that the observation of actions is less demanding in terms of cognitive load, leading to a more robust and less susceptible intervention to influence variables related to brain representation [73].

As far as the authors are concerned, this is the first randomized clinical trial that has studied the use of VR for NS-CNP and compared pain-related, motor and psychological outcomes compared to an exercise group in the short-term. Recent reviews have concluded that more research is needed regarding VR as a treatment of musculoskeletal disorders [24,26,65]. The present study matches the protocol of the reviewed articles where the treatment was delivered during four weeks of chronic pain, with similar immediate results [65]. The novelty of this research also relies on the use of two different free mobile applications with low budget VR goggles. This would significantly reduce the cost of treatment and, after further research on this potential and safe tool, health care professionals could take advantage of all the benefits that this therapy could mean for the patients. 

### 4.1. Clinical Implications

According to the obtained results, VR could be an effective tool for reducing the pain-related fear of movement and as effective as exercise in decreasing pain intensity or modulating pain. Putting its applicability into practice will be useful for patients with limited mobility and fear of movement as a previous step for active exercise and therapy, improving those aspects and increasing their internal locus of control and self-efficacy during the initial phases when motion is more limited, or pain is very severe. Given the cost and its effectiveness, it is worthy to note that a VR headset should be implemented in clinics for the patients who may benefit from this intervention, using these contemporary tools as useful interventions in conjunction with traditional treatments, especially since the new technologies are gaining ground on daily life activities. Furthermore, it could present collateral benefits such as enhancing their adherence to treatment and autonomy to practice at home. The authors also encourage further research on this topic so the mechanisms of VR and its applicability will be better understood in order to obtain more benefits for patients.

### 4.2. Study Limitations

This study presents several limitations. There was no placebo group, so we could not compare the non-specific effects of this therapy, similarly, there was no group that received a passive treatment, meaning both of our groups underwent active interventions which may explain our minimum differences between groups. In addition, the patients could not be blinded since they were aware of wearing an immersive VR device. Another limitation is that our results show the effects on a short-term period (3 months) so it would be interesting to see the long-term effects of this therapy. Moreover, motion sickness produced by virtual reality headsets was not taken into account and measuring it could influence its effects on the subjects of study. Furthermore, it is unclear if using different types of VR (semi-immersive or non-immersive), more adapted games specifically focused on muscle endurance or gradually introducing different grades of difficulty to the patients would have led to different results.

## 5. Conclusions

VR was not superior to exercise in improving pain intensity, CPM, ROM, neck disability, pain catastrophizing, fear-avoidance beliefs, PPT or anxiety in patients with NS-CNP. Kinesiophobia was the only variable that showed differences between VR and exercise at 3 months, with a decrease of almost 10 points in the VR group versus a decrease of less than 4 points in the exercise group. Therefore, immersive VR showed to be a more effective tool for reducing the pain-related fear of movement in NS-CNP.

## Figures and Tables

**Table 1:** Demographic and baseline characteristics from each group.

Variable	VR Group (*n* = 22) Mean ± SD (CI)	Control Group (*n* = 22) Mean ± SD (CI)	*p*-Value *
**Age (years)**	32.72 ± 11.63 (27.56–37.88)	26.68 ± 9.21 (22.59–30.76)	*p* = 0.063
**Male**	11 (50%) ^†^	10 (45.5%)	*p* = 0.763 ^‡^
**Female**	11 (50%) ^†^	12 (54.5%)	
**VAS**	4.97 ± 1.88 (4.20–5.74)	4.27 ± 1.3 (3.28–4.75)	*p* = 0.063
**CPM**	0.53 ± 0.74 (0.14–0.98)	0.67 ± 0.94 (0.14–0.97)	*p* = 0.667
**TS ^+^**	–0.54 ± 1.31 (−1.75–−0.23)	−1.00 ± 1.42 (−2.04–−0.71)	*p* = 0.380
**F/E(ROM)**	110.22 ± 19.19 (101.71–118.73)	116.13 ± 22.34 (106.22–126.04)	*p* = 0.352
**Lateroflexion (ROM)** **Rotation (ROM)**	79.54 ± 20.61 (70.40–88.68)	87.21 ± 17.96 (79.24–95.17)	*p* = 0.196
	114.10 ± 18.97 (105.69–122.52)	118.48 ± 15.19 (111.74–125.22)	*p* = 0.403
**NDI**	13.72 ± 6.68 (10.76–16.69)	14.09 ± 9.32 (9.95–18.22)	*p* = 0.883
**PCS**	17.36 ± 11.49 (12.26–22.45)	11.95 ± 9.39 (7.79–16.11)	*p* = 0.095
**Kinesiophobia**	22.90 ± 7.11 (19.75–26.06)	21.40 ± 6.63 (18.46–24.35)	*p* = 0.474
**FAQ**	28.25 ± 16.43 (21.58–34.91)	25.68 ± 13.02 (19.32–32.03)	*p* = 0.576
**PPT Right (kg/cm^2^)**	2.85 ± 1.59 (2.13–3.58)	2.95 ± 1.09 (2.45–3.44)	*p* = 0.828
**PPT Left (kg/cm^2^)**	3.88 ± 2.13 (2.93–4.83)	3.86 ± 1.69 (3.09–4.63)	*p* = 0.974
**PASS-20**	27.52 ± 20.52 (19.31–35.73)	26.59 ± 16.50 (18.57–34.61)	*p* = 0.870

* Comparison virtual reality group versus control group. ^†^ Absolute frequency and category percentage n (%) are shown. ^‡^ Pearson’s Chi-squared Test was used. ^+^ Median and interquartile range are represented for non-normal distributed variables. CI: Confidence Interval.

**Table 2:** Mean ± SD in primary and secondary outcomes and comparison between groups.

Variables	Times of Measurement	VR Group (*n* = 22) Mean ± SD (CI)	Control Group (*n* = 22) Mean ± SD (CI)	*p*-Value	Effect Size Cohen’s Method
**VAS**	Baseline	4.97 ± 1.88 (4.20–5.74)	4.27 ± 1.35 (3.57–4.97)	*p* = 0.063	0.27
	Post-treatment	2.67 ± 1.91 ^†^ (1.90–3.44)	3.11 ± 1.47 ^†^ (2.38–3.84)	*p* = 0.409	0.12
	Follow-up 1 Month	2.77 ± 2.04 ^†^ (1.91–3.63)	1.88 ± 1.74 ^†^ (1.07–2.70)	*p* = 0.137	0.31
	Follow-up 3 months	2.17 ± 1.99 ^†^ (1.24–3.10)	1.72 ± 2.09 ^†^ (0.84–2.61)	*p* = 0.484	0.10
**CPM (µs)**	Baseline	0.53 ± 0.74 (0.14–0.98)	0.67 ± 0.94 (0.14–0.97)	*p* = 0.667	0.70
	Post-treatment	0.85 ± 0.65 (0.39–1.27)	1.16 ± 1.11 (0.47–1.33)	*p* = 0.361	0.14
	Follow-up 1 Month	0.89 ± 0.91 (0.36–1.20)	0.75 ± 0.91 (0.23–1.04)	*p* = 0.682	0.06
	Follow-up 3 months	0.88 ± 0.79 (0.41–1.14)	0.62 ± 0.68 (0.18–0.89)	*p* = 0.335	0.15
**TS**	Baseline	−0.54 ± 1.31 (−1.75–−0.23)	−1.00 ± 1.42 (−2.04–−0.71)	*p* = 0.380	0.11
	Post-treatment	−0.75 ± 1.53 (−1.61–0.10)	−1.14 ± 1.49 (−1.89–−0.38)	*p* = 0.493	0.10
	Follow-up 1 Month	−0.37 ± 1.52 (−1.28–0.53)	−1.69 ± 1.65 (−2.48–−0.89)	*p* = 0.034 *	0.57
	Follow-up 3 months	−0.30 ± 1.97 (−5.42–4.81)	−3.54 ± 11.79 (−8.01–0.93)	*p* = 0.338	0.15
**F/E(ROM)**	Baseline	110.22 ± 19.19 (101.26–119.19)	116.13 ± 22.34 (107.17–125.09)	*p* = 0.352	0.15
	Post-treatment	112.01 ± 19.05 (103.19–120.83)	116.92 ± 21.84 (108.10–125.74)	*p* = 0.431	0.12
	Follow-up 1 Month	113.18 ± 24.13 (103.97–122.38)	117.63 ± 18.23 (108.43–126.84)	*p* = 0.494	0.10
	Follow-up 3 months	112.03 ± 23.99 (101.64–122.41)	117.60 ± 24.27 (107.22–127.99)	*p* = 0.448	0.11
**Lateroflexion (ROM)**	Baseline	79.54 ± 20.61 (70.96–88.12)	87.21 ± 17.96 (78.78–95.63)	*p* = 0.196	0.23
	Post-treatment	80.28 ± 22.25 (70.96–89.06)	89.07 ± 20.04 (79.96–98.18)	*p* = 0.181	0.26
	Follow-up 1 Month	82.85 ± 21.91 (74.25–91.46)	89.06 ± 16.94 (80.65–97.46)	*p* = 0.304	0.17
	Follow-up 3 months	84.93 ± 21.47 (76.46–93.40)	86.42 ± 16.79 (78.14–94.69)	*p* = 0.801	0.05
**Rotation (ROM)**	Baseline	114.10 ± 18.97 (107.66–123.24)	118.48 ± 15.19 (111.05–125.91)	*p* = 0.403	0.08
	Post-treatment	117.31 ± 23.64 (108.37–126.26)	122.07 ± 15.49 (113.54–130.60)	*p* = 0.441	0.11
	Follow-up 1 Month	122.15 ± 19.42 (114.10–130.19)	122.34 ± 16.17 (114.68–130.01)	*p* = 0.971	0.05
	Follow-up 3 months	121.90 ± 18.49 (114.21–129.58)	124.30 ± 15.55 (116.97–131.63)	*p* = 0.650	0.07
**NDI**	Baseline	13.72 ± 6.68 (9.66–16.72)	14.09 ± 9.32 (10.64–17.54)	*p* = 0.883	0.06
	Post-treatment	6.90 ± 6.28 (4.33–9.47)	7.45 ± 5.36 (4.94–9.96)	*p* = 0.759	0.06
	Follow-up 1 Month	5.57 ± 6.32 (2.97–8.16)	5.95 ± 5.43 (3.41–8.49)	*p* = 0.832	0.05
	Follow-up 3 months	4.95 ± 6.60 (2.44–7.46)	5.77 ± 4.67 (3.31–8.22)	*p* = 0.640	0.07
**PCS**	Baseline	17.36 ± 11.49 (12.20–21.41)	11.95 ± 9.39 (7.45–16.45)	*p* = 0.095	
	Post-treatment	8.52 ± 9.77 (4.97–12.07)	6.40 ± 5.98 (2.94–9.87)	*p* = 0.395	
	Follow-up 1 Month	6.33 ± 10.06 (2.89–9.77)	3.72 ± 4.72 (0.37–7.08)	*p* = 0.280	
	Follow-up 3 months	4.95 ± 8.08 (1.31–8.58)	4.86 ± 8.40 (1.31–8.41)	*p* = 0.136	
**Kinesiophobia**	Baseline	22.90 ± 7.11 (19.74–25.87)	21.40 ± 6.63 (18.41–24.40)	*p* = 0.474	0.09
	Post-treatment	18.90 ± 10.73 (14.84–22.96)	18.36 ± 7.48 (14.39–22.33)	*p* = 0.848	0.05
	Follow-up 1 Month	14.85 ± 10.08 ^†^ (10.90–18.08)	17.54 ± 7.75 (13.68–21.40)	*p* = 0.331	0.16
	Follow-up 3 months	12.09 ± 7.77 ^†^ (8.86–15.33)	17.50 ± 6.89 (14.33–20.66)	*p*< 0.05 *	0.65
**FAQ**	Baseline	28.25 ± 16.43 (21.58–34.91)	25.68 ± 13.02 (19.32–32.03)	*p* = 0.576	
	Post-treatment	20.55 ± 16.78 (13.18–27.91)	18.81 ± 15.84 (11.79–25.84)	*p* = 0.733	
	Follow-up 1 Month	18.95 ± 20.19 (11.31–26.58)	15.84 ± 13.24 (8.58–23.14)	*p* = 0.558	
	Follow-up 3 months	12.30 ± 13.48 (6.00–18.59)	16.59 ± 14.32 (10.58–22.59)	*p* = 0.325	
**PPT Right (Kg/cm^2^)**	Baseline	2.85 ± 1.59 (2.38–3.64)	2.95 ± 1.09 (2.35–3.55)	*p* = 0.828	0.05
	Post-treatment	3.09 ± 1.33 (2.56–3.61)	3.12 ± 0.90 (2.62–3.62)	*p* = 0.932	0.05
	Follow-up 1 Month	3.43 ± 1.44 (2.83–4.03)	3.35 ± 1.14 (2.78–3.92)	*p* = 0.854	0.05
	Follow-up 3 months	3.53 ± 1.27 (2.96–4.01)	3.28 ± 1.17 (2.74–3.83)	*p* = 0.525	0.09
**PPT Left (Kg/cm^2^)**	Baseline	3.38 ± 2.13 (3.20–4.94)	3.86 ± 1.69 (3.01–4.71)	*p* = 0.974	0.06
	Post-treatment	4.77 ± 2.38 (3.83–5.70)	4.07 ± 1.70 (3.15–4.98)	*p* = 0.284	0.18
	Follow-up 1 Month	3.55 ± 0.96 (3.04–4.05)	3.58 ± 1.25 (3.09–4.08)	*p* = 0.920	0.05
	Follow-up 3 months	3.78 ± 0.97 (3.29–4.27)	3.40 ± 1.17 (2.92–3.88)	*p* = 0.266	0.19
**PASS-20**	Baseline	27.52 ± 20.52 (19.31–35.73)	26.59 ± 16.50 (18.57–34.61)	*p* = 0.870	0.05
	Post-treatment	17.33 ± 16.71 (10.97–23.69)	17.86 ± 11.85 (11.65–24.07)	*p* = 0.905	0.05
	Follow-up 1 Month	14.09 ± 17.44 (7.48–20.70)	16.04 ± 12.20 (9.59–22.50)	*p* = 0.672	0.07
	Follow-up 3 months	12.33 ± 16.09 (6.23–18.43)	15.90 ± 11.30 (9.94–21.87)	*p* = 0.402	0.13

* statistically significant for time factor (*p* < 0.05) from baseline intragroup comparison. ^†^ Welch’s T-test was used. (CI): Confidence Interval.
